# Genome-wide identification of *JAZ* gene family members in autotetraploid cultivated alfalfa (*Medicago sativa* subsp. *sativa*) and expression analysis under salt stress

**DOI:** 10.1186/s12864-024-10460-6

**Published:** 2024-06-26

**Authors:** Wei Yan, Xueming Dong, Rong Li, Xianglong Zhao, Qiang Zhou, Dong Luo, Zhipeng Liu

**Affiliations:** State Key Laboratory of Herbage Improvement and Grassland Agro-ecosystems, Key Laboratory of Grassland Livestock Industry Innovation, Ministry of Agriculture and Rural Affairs, Engineering Research Center of Grassland Industry, Ministry of Education, College of Pastoral Agriculture Science and Technology, Lanzhou University, Lanzhou, 730000 People’s Republic of China

**Keywords:** Autotetraploid cultivated alfalfa, *JAZ* genes, Expression analysis, Salt stress

## Abstract

**Background:**

Jasmonate ZIM-domain (JAZ) proteins, which act as negative regulators in the jasmonic acid (JA) signalling pathway, have significant implications for plant development and response to abiotic stress.

**Results:**

Through a comprehensive genome-wide analysis, a total of 20 members of the *JAZ* gene family specific to alfalfa were identified in its genome. Phylogenetic analysis divided these 20 *MsJAZ* genes into five subgroups. Gene structure analysis, protein motif analysis, and 3D protein structure analysis revealed that alfalfa *JAZ* genes in the same evolutionary branch share similar exon‒intron, motif, and 3D structure compositions. Eight segmental duplication events were identified among these 20 *MsJAZ* genes through collinearity analysis. Among the 32 chromosomes of the autotetraploid cultivated alfalfa, there were 20 *MsJAZ* genes distributed on 17 chromosomes. Extensive stress-related *cis*-acting elements were detected in the upstream sequences of *MsJAZ* genes, suggesting that their response to stress has an underlying function. Furthermore, the expression levels of *MsJAZ* genes were examined across various tissues and under the influence of salt stress conditions, revealing tissue-specific expression and regulation by salt stress. Through RT‒qPCR experiments, it was discovered that the relative expression levels of these six *MsJAZ* genes increased under salt stress.

**Conclusions:**

In summary, our study represents the first comprehensive identification and analysis of the *JAZ* gene family in alfalfa. These results provide important information for exploring the mechanism of *JAZ* genes in alfalfa salt tolerance and identifying candidate genes for improving the salt tolerance of autotetraploid cultivated alfalfa via genetic engineering in the future.

**Supplementary Information:**

The online version contains supplementary material available at 10.1186/s12864-024-10460-6.

## Background

Alfalfa (*Medicago sativa* subsp. *sativa*), which is hailed as the “queen of forage” because of its ease of harvesting, digestibility by livestock, high protein content, and high yield, is a crop cultivated globally and covers an extensive planting area of approximately 30 million hectares, with an annual production reaching approximately 450 million tons [1]. Alfalfa is widely grown in the United States, Netherlands, and other countries, and it is the fourth most grown crop in the United States, with an annual cultivation area of 8.5–9.3 million hectares [2]. In China, alfalfa is mainly planted in northern regions, including provinces such as Gansu and Xinjiang, and its total cultivated area is approximately four million hectares [3]. XinJiangDaYe, which is an autotetraploid (2n = 4x = 32) alfalfa cultivar characterized by leaves larger than those of other alfalfa cultivars, is a unique local alfalfa variety in China and is widely grown in Xinjiang and other provinces [4]. However, improper irrigation, improper fertilization, and industrial pollution can increase soil salinity, which has resulted in more than one billion hectares of land globally being affected by salt [5, 6]. High salinity in the soil typically arises from elevated concentrations of Na^+^ and Cl^−^ ions in the soil solution, leading to hyperosmotic conditions that hinder the absorption of water and nutrients by plants from the soil [7, 8]. The majority of plants are glycophytes, and their growth and productivity are adversely affected by soil salt stress. Alfalfa, a forage cultivated worldwide, is inevitably affected by salt stress. Therefore, the molecular breeding of alfalfa by genetic engineering is an effective method for improving its salt tolerance.

Plant hormones, mainly abscisic acid (ABA), gibberellin (GA), auxin (IAA), ethylene (ET), and jasmonic acid (JA), are crucial endogenous substances that play a vital role in regulating plant physiology and molecular processes, and they are essential for plant growth and development [9]. Jasmonates (JAs) and oxylipin derivatives are lipid plant hormones that can promote plant senescence, inhibit seedling growth, and improve the ability to adapt to abiotic and abiotic stresses [10–13]. When plants face biotic or abiotic stresses, they produce a large amount of JA to enhance their resistance [14–18]. For example, JA and methyl jasmonate (MeJA) induce the accumulation of protease inhibitors to resist insect attacks on plants [19–21]. Endogenous JA enhances the salt tolerance of tomatoes by activating enzymatic and nonenzymatic antioxidants to maintain the homeostasis of reactive oxygen species [22]. When plants are subjected to stress, JA binds to isoleucine (Ile) to form a JA-Ile conjugate, which is capable of binding and activating the ubiquitin ligase complex (SCF^COI1^) and can promote JAZ protein degradation, thereby increasing the content of JA in plants [23].

JAZ proteins are important regulators of the JA signalling pathway and function as negative regulators to suppress the activity of related transcription factors [24]. JAZ proteins possess two crucial domains: the TIFY domain, which is also known as the ZIM domain and is located at the N-terminus, and the Jas domain, which is also known as the CCT_2 domain and is located at the C-terminus [25]. The TIFY domain in JAZ proteins contains a conserved sequence known as the TIFY motif, which typically follows the pattern TIF[F/Y] XG [26]. This motif is important for protein‒protein interactions and plays a role in the regulation of JA signalling. This domain interacts with the NOVEL INTERACTOR OF JAZ (NINJA) protein, which has an ERF-associated amphiphilic repression (EAR) motif, leading to the recruitment of TOPLESS (TPL) proteins, and this recruitment further promotes the transcriptional repression of JA responses, effectively inhibiting JA signal activation [27–29]. However, the Jas domain contains a conserved sequence referred to as SLX_2_FX_2_KRX_2_RX_5_PY. This sequence is crucial for binding to upstream coronavirus toxin-insensitive 1 (COI1), which is necessary for JAZ protein degradation [30]. JAZ protein degradation is an important step in the activation of the JA signalling pathway. *JAZ* gene family members have been classified into five subgroups (groups I - V) according to their phylogenetic relationships [31]. *Arabidopsis* has 13 *JAZ* gene family members (*AtJAZ1*–*AtJAZ13*) clustered into five groups, namely, group I (*AtJAZ1*, *AtJAZ2*, *AtJAZ5*, and *AtJAZ6*), group II (*AtJAZ11* and *AtJAZ12*), group II (*AtJAZ10*), group III (*AtJAZ7*, *AtJAZ8*, and *AtJAZ13*), and group III (*AtJAZ3*, *AtJAZ4*, and *AtJAZ9*) [25, 30].

To date, numerous studies have provided evidence indicating the involvement of *JAZ* gene family members in the response to salt stress. The overexpression of *GhJAZ2* significantly increased the sensitivity of transgenic cotton plants to salt stress [32]. The transcriptional regulator OsJAZ9 forms transcriptional regulatory complexes with OsNINJA and OsbHLH in rice, playing a crucial role in finely regulating the expression of endogenous JA response genes associated with salt stress tolerance [33]. The overexpression of *GsJAZ2* from *Glycine soja* enhances the adaptability of *Arabidopsis* plants to salt stress [34]. Wheat enhances the expression of *JAZ* genes to inhibit the production of JA in stressed environments, thus inhibiting the metabolic processes controlled by JA and GA and improving adaptability to salt stress [35]. Additionally, when plants are not experiencing salt stress, the JAZ protein inhibits the activity of DNA-binding transcription factors that regulate the expression of genes involved in the JA response, ultimately controlling the levels of JA in plants; however, when plants are under salt stress, JAZ interacts with COI1 on JA molecules, the complex is recognized by SCF^COI1^ and degraded by the 26 S proteolytic pathway, and the released transcription factor activates the transcription of JA response genes, thereby increasing the content of JA in plants [29, 36].

To date, research on the salt stress-related functions of the *JAZ* gene family has been reported in numerous plant species, including sweet potato [37], *Sorghum bicolor* [38], and turnip [39]. However, research on the salt stress response of *JAZ* genes in autotetraploid cultivated alfalfa is scarce. The genome of autotetraploid alfalfa and the transcriptome of salt-stressed alfalfa have been published, providing data for the analysis of the molecular structure and role of the alfalfa *JAZ* gene family under salt stress [2, 40]. Here, the genome-wide identification of alfalfa *JAZ* genes was performed using bioinformatics technology, and their physicochemical properties, evolutionary relationships, sequence features, three-dimensional structures, synteny, chromosome maps, and *cis*-acting elements were analysed. Additionally, we investigated the expression patterns of *MsJAZ* genes in different tissues and under salt stress conditions. These findings will provide important foundations for molecular breeding of autotetraploid cultivated alfalfa with improved salt tolerance through genetic engineering.

## Materials and methods

### Genome-wide identification and Gene Ontology (GO) analysis of alfalfa *JAZ* genes

The alfalfa genome files used were obtained from XinJiangDaYe, and these files were obtained from the figshare projects (https://figshare.com/projects/whole_genome_sequencing_and_assembly_of_Medicago_sativa/66380). The Hidden Markov Model (HMM) files for two domains of the JAZ protein, the TIFY domain (PF06200) and the JAS domain (PF09425), were downloaded from the Pfam protein family database (http://pfam.sanger.ac.uk/). The identification of JAZ proteins in alfalfa was performed in three steps. First, the downloaded HMM files were used to search the alfalfa protein file using HMMER 3.2.1, with the E-value set to ≤ 0.01. Then, each protein sequence that was searched was examined using the NCBI-CDD database (https://www.ncbi.nlm.nih.gov/Structure/cdd/wrpsb.cgi) to identify any conserved domains it contained. Sequences that did not possess both of the desired conserved domains were excluded from further analysis. Finally, the redundant sequences were eliminated by the decrease redundancy tool (https://web.expasy.org/decrease_redundancy/) with default parameters. The remaining sequences were identified as MsJAZ proteins and used for further analysis. The physicochemical properties of the identified MsJAZ proteins were determined using the ProtParam tool (https://web.expasy.org/protparam/). Subcellular localization prediction for all MsJAZ proteins was performed by WoLF PSORT (https://wolfpsort.hgc.jp/). Additionally, all *MsJAZ* genes were subjected to GO annotation analysis using the website of the online database eggnog-mapper (http://eggnog-mapper.embl.de/).

### Phylogeny, gene structure, and conserved motif analysis

To study the evolutionary relationships of *JAZ* genes in alfalfa (*n* = 20), *Arabidopsis* (*n* = 13), *Medicago truncatula* (model plant of legumes, *n* = 14), and rice (*n* = 15), an evolutionary tree was created. First, a multiple sequence alignment for all the JAZ proteins was performed via ClustalW (http://www.clustal.org/clustal2/) [41]. Then, four methods, namely, neighbour joining (NJ), maximum likelihood (ML), minimum evolution (ME), and unweighted pair group method with arithmetic mean (UPGMA), were used to create the evolutionary tree via MEGA 7 software (https://www.megasoftware.net/). Based on the grouping method of JAZ proteins in *Arabidopsis*, all JAZ proteins on the evolutionary tree were categorized into groups. The gene structures of all *MsJAZ* genes were analysed using the Gene Structure Display Server (GSDS) (http://gsds.cbi.pku.edu.cn/). In addition, conserved motifs of all MsJAZ proteins were identified and analysed via the MEME 5.5.3 online tool (https://meme-suite.org/meme/), with a minimum motif length of 20, a maximum motif length of 100, a maximum number of motifs of 10, and a repetition number of 0 or 1 [42].

### 3D structure analysis of MsJAZ proteins

The 3D structure of a protein is the basis for its functional role. The secondary structures of all MsJAZ proteins, including α-helices, extended strands, β-turns, and random coils, were predicted and analysed using SOPMA.(https://npsa.lyon.inserm.fr/cgi-bin/npsa_automat.pl?page=/NPSA/npsa_sopma.html) The quality of the predicted models was assessed using the Global Model Quality Estimation (GMQE) value.

### Gene duplication analysis and chromosomal mapping

TBtools software was used to identify duplication events in the 20 *MsJAZ* genes through collinearity analysis [43]. KaKs_Calculator 2.0 was used to calculate the nonsynonymous replacement rate (Ka), synonymous replacement rate (Ks), and Ka/Ks of identified duplicate gene pairs [44]. Additionally, we performed collinearity analysis between alfalfa and *Arabidopsis*, *Medicago truncatula*, and rice *JAZ* genes. The chromosomal distribution of *MsJAZ* genes in alfalfa was analysed and visualized using MapGene2Chrome (http://mg2c.iask.in/mg2c_v2.1/) [45].

### Analysis of promoter *cis*-acting element

The identification and analysis of *cis*-acting elements in the upstream regions of *MsJAZ* genes can provide evidence of their potential ability to respond to salt stress. First, TBtools software was used to extract the upstream 2000 bp sequences of all *MsJAZ* genes. Then, PlantCARE (https://bioinformatics.psb.ugent.be/webtools/plantcare/html/) was used to identify and analyse the *cis*-acting elements in these sequences [46]. All identified *cis*-acting elements were categorized based on their functions [47].

### Expression analysis of *MsJAZ* genes in different tissues

To determine the expression levels of *MsJAZ* genes in different tissues, transcriptome data for alfalfa from various tissues were downloaded from the MODMS (https://modms.lzu.edu.cn). These tissues included leaves, flowers, pre-elongated stems, elongated stems, roots, and nodules. Then, we visualized these expression levels with TBtools software. Additionally, correlation analysis of *MsJAZ* gene expression across these six tissues was performed using Corrplot software in R.

### Expression analysis of *MsJAZ* genes in response to salt stress

To determine the expression patterns of *MsJAZ* genes under salt stress, RNA-Seq data from alfalfa plants under salt stress (SRR7160313-SRR7160357) generated by our laboratory were utilized. First, a local BLAST alignment was performed against this transcriptome dataset using the nucleotide sequences of *MsJAZ* genes as queries [48]. Then, TBtools software was used to analyse and visualize these data. Additionally, the expression patterns of *MsJAZ* genes under salt stress were clustered by the Mfuzz package in R.

### Plant material and salt stress treatment

Alfalfa (XinJiangDaYe) seeds were germinated and subjected to salt stress as previously described [49]. Root tips of alfalfa plants were collected at different time points (0, 1, 3, 6, 12, and 24 h) after salt stress, as well as at 1 and 12 h after stress removal. All collected materials were immediately flash-frozen in liquid nitrogen and stored at -80 °C for preservation.

### RT‒qPCR analysis

We selected six *MsJAZ* genes whose expression increased in response to salt treatment and used them to verify the reliability of the RNA-Seq data through RT‒qPCR experiments. The experimental methods, reagents, and instruments used for the RT‒qPCR experiments were consistent with those in previously described methods [47]. The specific primers used for the RT‒qPCR of the six selected *MsJAZ* genes were designed with Primer3Plus (https://www.primer3plus.com/) (Table S1). There were three replicates per treatment and three replicates per reaction. Finally, we calculated the relative expression levels of six selected *MsJAZ* genes via the 2^−ΔΔCT^ method.

### Subcellular localization analysis

To further investigate the *JAZ* genes whose expression significantly increased under salt stress, we selected *MsJAZ1* for subcellular localization experiment. We amplified the full-length coding sequence of this gene without stop codons and cloned it into the 5518-pSuper1300-eGFP vector, generating the 5518-pSuper1300-MsJAZ1-eGFP fusion plasmid. The specific primers used for amplification are listed in Table S1. The successfully constructed plasmid and the control vector containing only eGFP were then transformed into Agrobacterium tumefaciens GV3101 for transient expression in tobacco leaves. After one day of incubation in darkness, the leaves were transferred to a growth chamber under light conditions for another day. The cell nuclei were stained with DAPI, and the fluorescence signal of GFP was captured using a laser confocal microscope.

## Results

### Genome-wide identification and Gene Ontology (GO) analysis of the *JAZ* genes

**Fig. 1:**
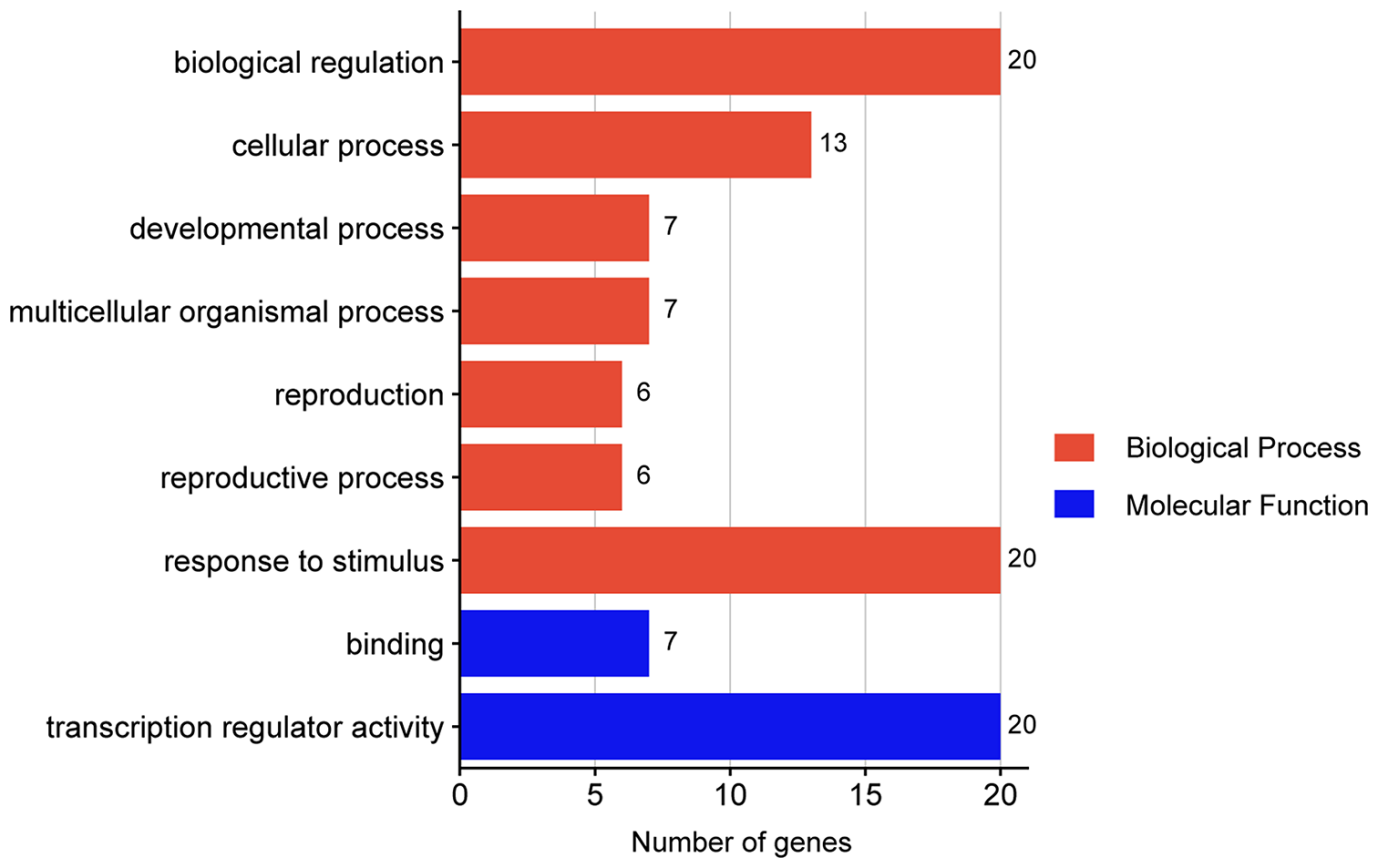
Gene Ontology (GO) annotation results for *MsJAZ* genes. Different functional categories are represented by different colours

After removing the sequences lacking both the TIFY domain and JAS domain and redundant sequences, 20 alfalfa *JAZ* gene family members were identified, and gene sequence and protein sequence information for these genes is provided in Table S2 and Table S3. Then, we studied the physicochemical properties of MsJAZ proteins using ExPASy ProtParam, and detailed information is provided in Table S3. The length of the MsJAZ proteins varied from 119 (MsJAZ20) to 429 (MsJAZ16) amino acids, the molecular weight varied from 13311.39 (MsJAZ20) to 46232.77 (MsJAZ16) Da, and the PIs varied from 4.97 (MsJAZ20) to 9.61 (MsJAZ1). All the JAZ proteins were considered to be unstable, soluble, and hydrophilic, as indicated by their instability index of greater than 40 and grand average of hydropathicity (GRAVY) of less than 0. Additionally, based on subcellular localization prediction, all MsJAZ proteins were found to be predominantly localized within the nucleus, while MsJAZ5, MsJAZ9, and MsJAZ10 may also play functional roles in the cell membrane (Table S3). Gene Ontology (GO) analysis revealed that the functions of all the *MsJAZ* genes could be divided into biological processes and molecular functions, and the detailed information is shown in Fig. 1 and Table S4. The biological process category included seven terms, namely, “biological regulation”, “cellular process”, “developmental process”, “multicellular organismal process”, “reproduction”, “reproductive process”, and “response to stimulus”, and 20 *MsJAZ* genes were assigned to “biological regulation” and “response to stimulus”. The molecular functional categories included “binding” and “transcriptional regulator activity”, with 7 and 20 *MsJAZ* genes assigned to “binding” and “transcription regulator activity”, respectively.

### Phylogeny, gene structure, and conserved motif analysis

**Fig. 2:**
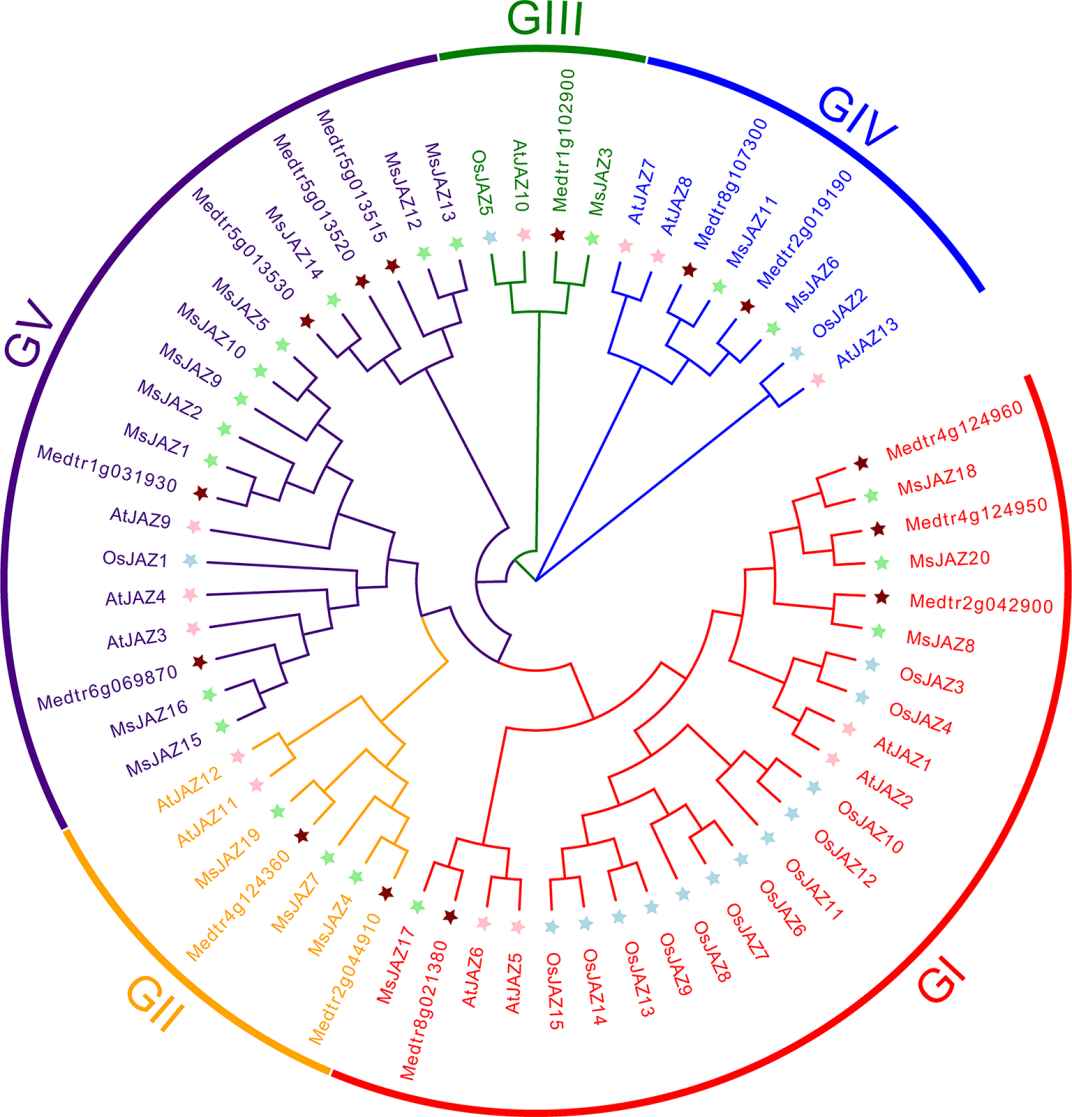
Phylogenetic relationships among JAZ proteins in alfalfa, *Arabidopsis*, *M. truncatula*, and rice. The phylogenetic tree was created by the NJ method. Different groups are indicated by different colours, and stars of different colours represent alfalfa, *Arabidopsis*, *M. truncatula*, and rice JAZ proteins

**Fig. 3:**
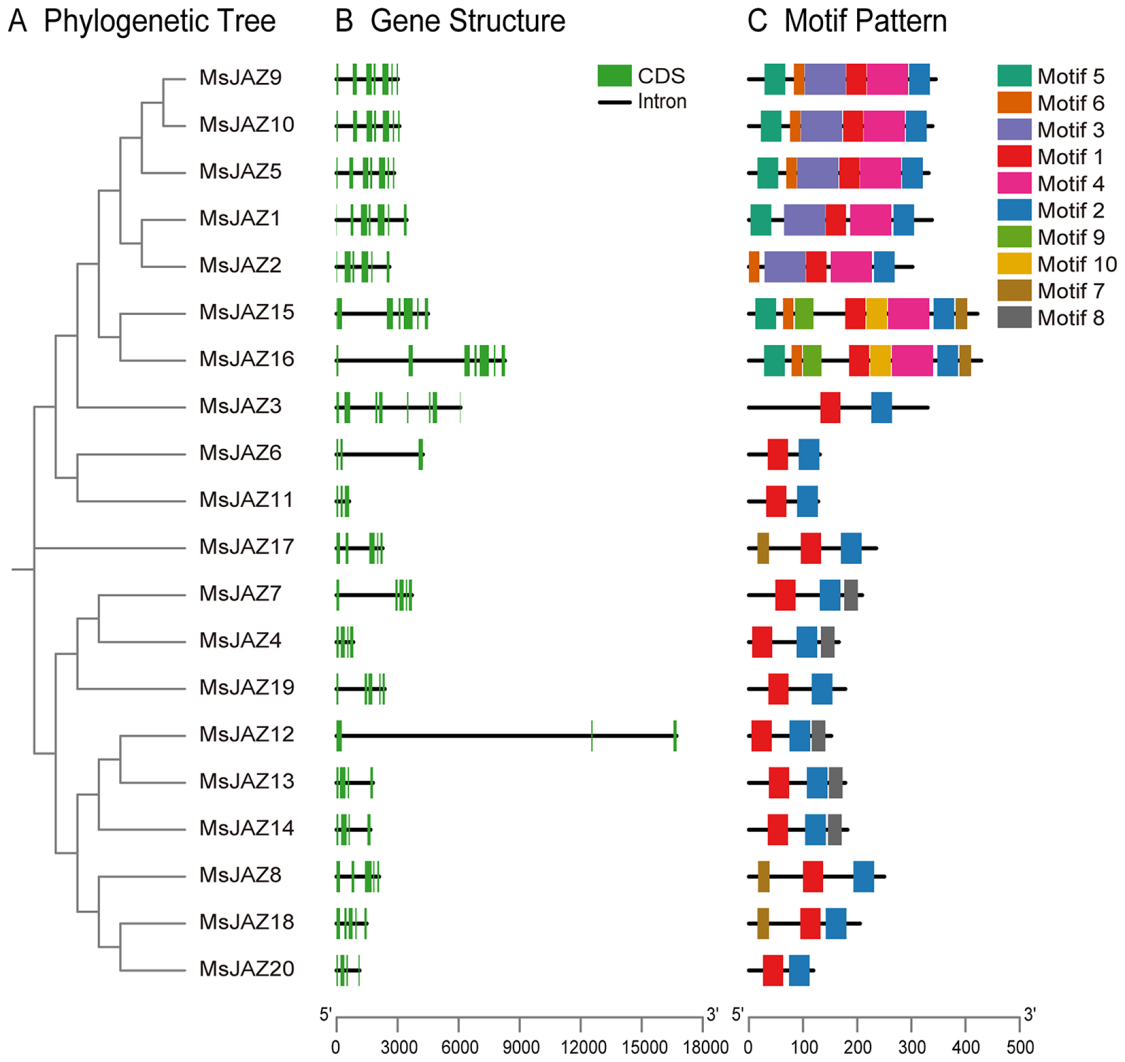
Phylogenetic relationships, gene structures, and conserved motifs. (**A**) Phylogenetic relationships of *MsJAZ* genes. The phylogenetic tree was created by the NJ method. (**B**) Gene structures of *MsJAZ* genes. The green boxes and black lines represent CDSs and introns, respectively. (**C**) Motif patterns of MsJAZ proteins. Boxes of different colours represent different motifs

To study the phylogenetic relationships of the JAZ proteins in alfalfa, *Arabidopsis*, *Medicago truncatula*, and rice, a phylogenetic tree was created via the neighbour–joining (NJ) method (bootstrap = 1000) (Fig. 2). All JAZ proteins on the phylogenetic tree were divided into 5 subgroups (GI–GIV) according to the classification of JAZ proteins in *Arabidopsis* (Figs. 2 and 3A). Among these groups, Group V had the greatest number of MsJAZ proteins (*n* = 10), and group III contained the smallest number of MsJAZ proteins (only one). Phylogenetic trees were constructed using ML, ME, and UPGMA methods, and these results showed similar groupings to those of the phylogenetic tree constructed using the NG method (Figs. S1,S2 and S3). The gene structures of all *MsJAZ* genes were analysed by GSDS, which showed that the number of coding sequences (exons) of the *MsJAZ* genes ranged from 3 to 8, among which *MsJAZ3* had the largest number of exons (*n* = 8), while *MsJAZ6*, *MsJAZ11*, and *MsJAZ12* had the smallest number of exons (*n* = 3) (Fig. 3B). Similarly, the number of exons in *MsJAZ* genes belonging to the same subgroup was similar. We used MEME to identify conserved motifs in MsJAZ proteins and their distribution, and detailed information on all motifs is shown in Table S5. JAZ proteins clustered in the same subgroup had motifs with similar numbers and types, indicating that they had similar functions (Fig. 3C). In addition, all MsJAZ proteins possessed motif 1 and motif 2.

### Secondary structure and 3D structure analyses of MsJAZ proteins

**Fig. 4:**
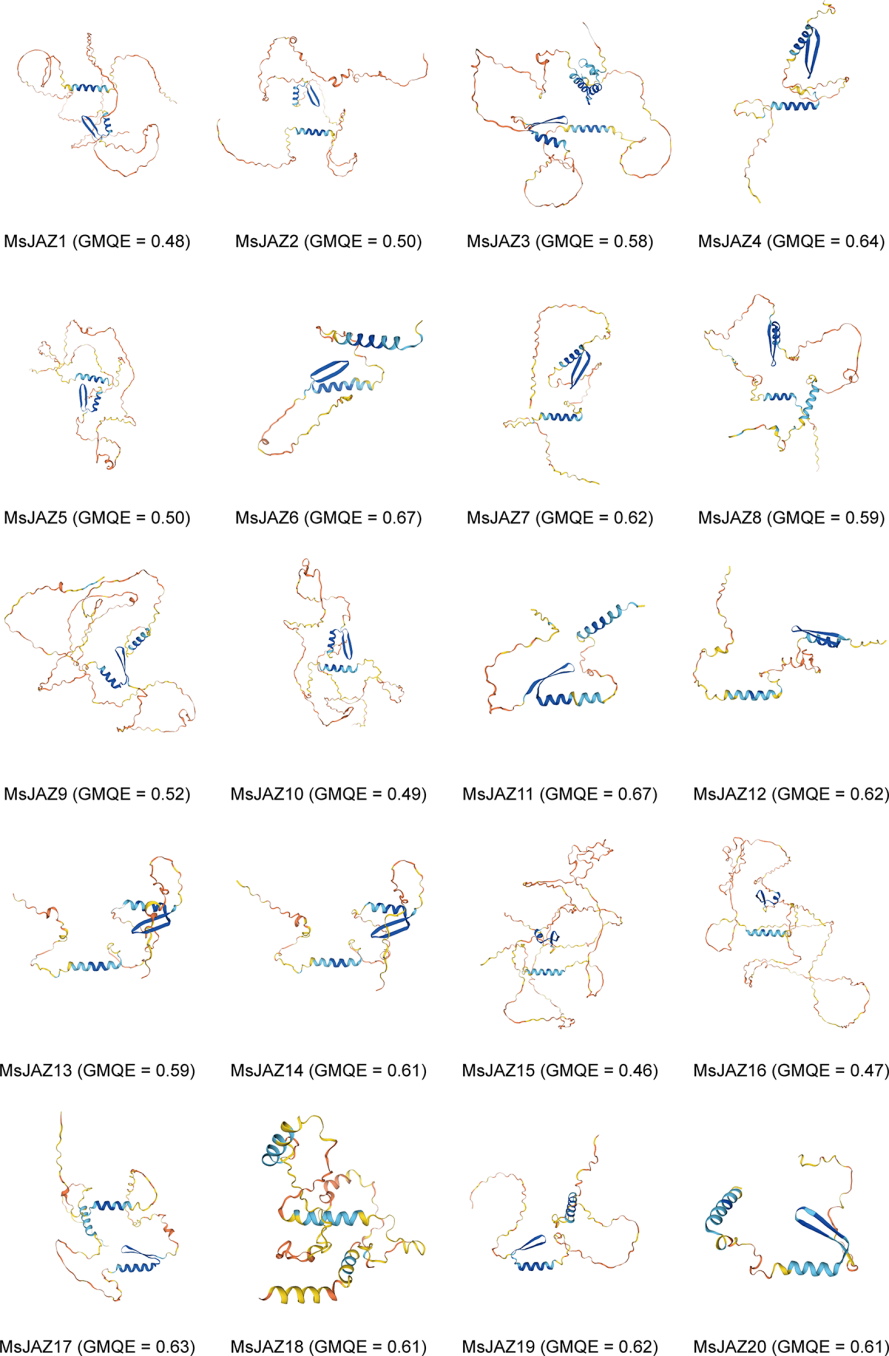
Predicted 3D structures of MsJAZ proteins. The quality of these predicted models was assessed using the GMQE value

The physical structures of proteins play important roles in their physiological and biochemical functions. Therefore, we predicted and analysed the secondary structures and 3D structures of all the MsJAZ proteins. The secondary structures of the MsJAZ proteins were determined by SOPMA, and the results showed that among all the MsJAZ proteins, those containing random coils comprised the largest proportion (51.38–72.19%), followed by those containing α-helices (10.66–28.91%), extended strands (5.15–18.49%), and β-turns (1.82–4.88%) (Table S6). SWISS-MODEL was used to predict the 3D structures of these proteins, and the GMQE value was used to evaluate the quality of these models (Fig. 4). The GMQE values of all the models were > 0.46, showing that these predicted 3D structures were reliable. These 3D structures were mainly composed of random coils and α-helices, which was consistent with the findings of the secondary structure analysis. Random coils existed in all the JAZ polypeptide chains and were the most widely distributed structural elements in the JAZ polypeptide chains. In addition, the 3D structures of the MsJAZ proteins on closely related evolutionary branches were similar, suggesting that these proteins may share similar physiological functions.

### Synteny analysis and chromosomal mapping

**Fig. 5:**
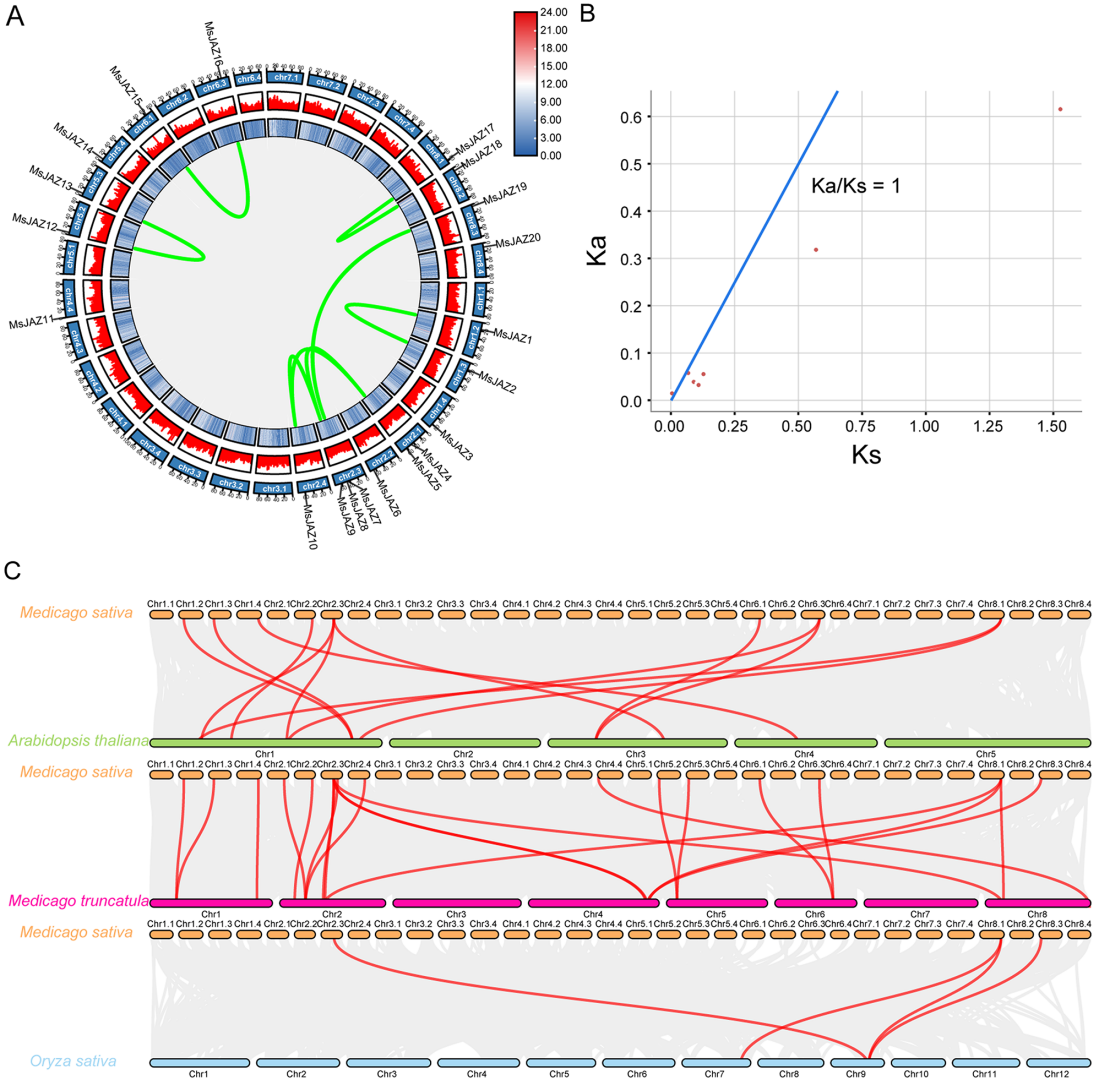
Synteny analysis of *JAZ* genes. (**A**) Synteny analysis of *JAZ* genes in the alfalfa genome. The grey lines in the central background represent all gene duplication events in the alfalfa genome. The red lines in the centre represent duplication events of *JAZ* genes. The outer circles in blue and red represent gene density. (**B**) Ka/Ks ratios of duplicated pairs of *MsJAZ* genes. The blue line indicates Ka/Ks = 1. (**C**) Synteny analysis of *JAZ* genes between alfalfa and *Arabidopsis*, *Medicago truncatula*, and rice. The grey lines in the background represent all gene duplication events. The red lines represent duplication events of *JAZ* genes

**Fig. 6:**
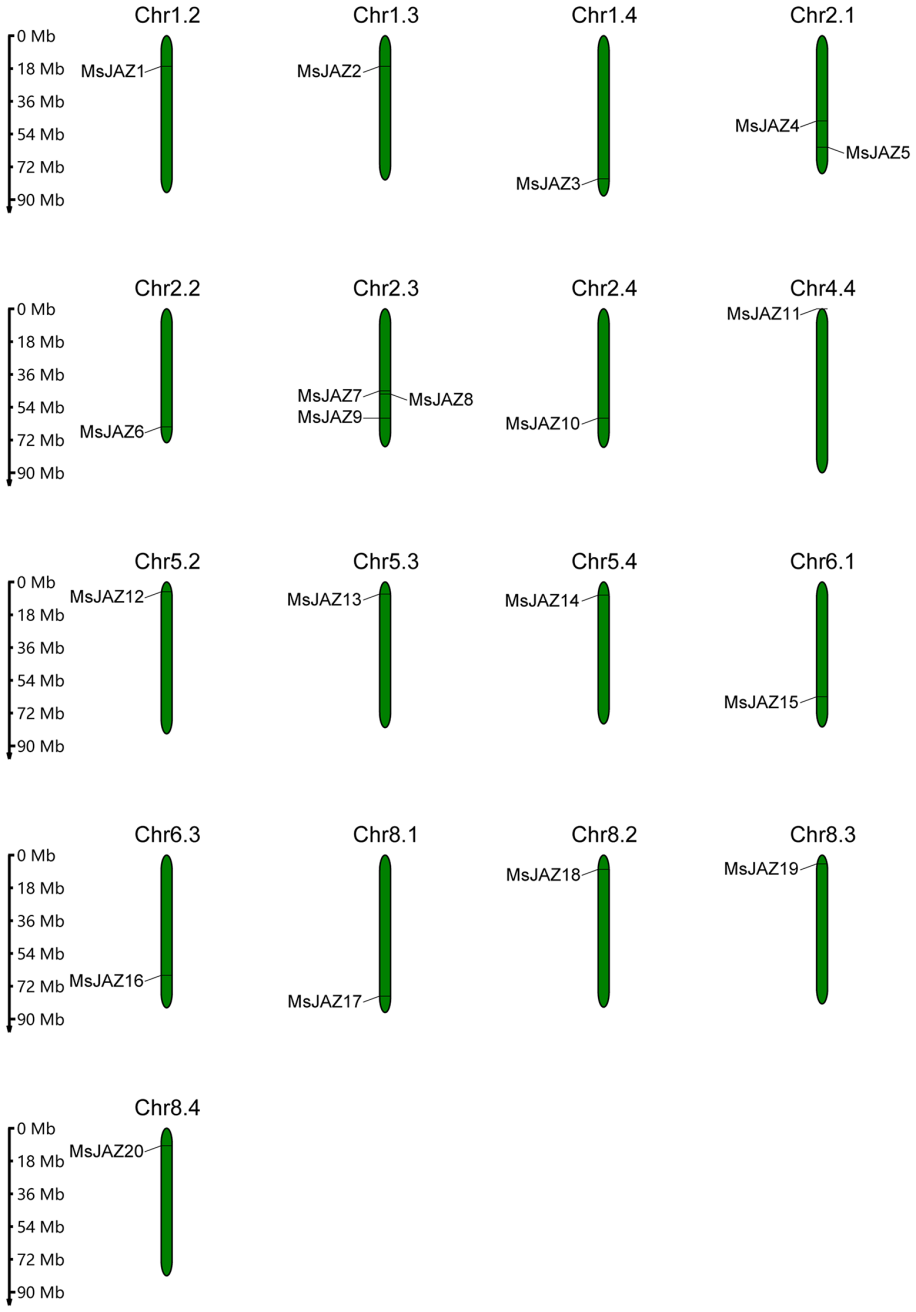
Chromosomal mapping of 20 *MsJAZ* genes

To identify gene duplication events, we performed collinearity analysis among these 20 *MsJAZ* genes. Gene replication events include segmental duplication, which refers to replication events between chromosomes, and tandem duplication, which refers to replication events within chromosomes. In our study, 8 pairs of segmental duplications in *MsJAZ* genes (*MsJAZ1*/*MsJAZ2*, *MsJAZ5*/*MsJAZ9*, *MsJAZ5*/*MsJAZ10*, *MsJAZ9*/*MsJAZ10*, *MsJAZ7*/*MsJAZ19*, *MsJAZ12*/*MsJAZ13*, *MsJAZ15*/*MsJAZ16*, and *MsJAZ17*/*MsJAZ18*) were identified, but no tandem duplications were detected (Fig. 5A). To understand the evolutionary constraints of *JAZ* gene duplication in alfalfa, the Ka, Ks, and Ka/Ks ratios for each segmental duplicated *JAZ* gene pair were analysed (Fig. 5B and Table S7). The Ka/Ks ratio of *MsJAZ1*/*MsJAZ2* was greater than one (Ka/Ks = 3.201219), and those of the others were less than one, suggesting that purifying selection was the primary factor affecting the evolution of alfalfa *JAZ* genes. To investigate the role of *JAZ* genes in the direct evolution of different species, three comparative synteny maps were generated using TBtools software. These maps included comparisons between alfalfa and three reference species: *Arabidopsis thaliana*, *Medicago truncatula*, and *Oryza sativa* (Fig. 5C and Table S8). There were 12, 21, and 4 syntenic gene pairs between alfalfa and the three reference species *Arabidopsis thaliana*, *Medicago truncatula*, and *Oryza sativa*, respectively. Moreover, *MsJAZ8* and *MsJAZ17* had homologous gene pairs among these three species. Additionally, we analysed the chromosomal distribution of 20 *MsJAZ* genes in autotetraploid cultivated alfalfa, as shown in Fig. 6. The results revealed that the *MsJAZ* genes were distributed on various chromosomes of alfalfa. Specifically, there was only one *MsJAZ* gene present on chromosomes Chr1.2, Chr1.3, Chr1.4, Chr2.2, Chr2.4, Chr4.4, Chr5.2, Chr5.3, Chr5.4, Chr6.1, Chr6.3, Chr8.1, Chr8.2, Chr8.3, and Chr8.4. However, on chromosome Chr2.1, there were two *MsJAZ* genes (*MsJAZ4* and *MsJAZ5*), and on chromosome Chr2.3, there were three *MsJAZ* genes (*MsJAZ7*, *MsJAZ8*, and *MsJAZ9*).

### Analysis of promoter *cis*-acting elements

**Fig. 7:**
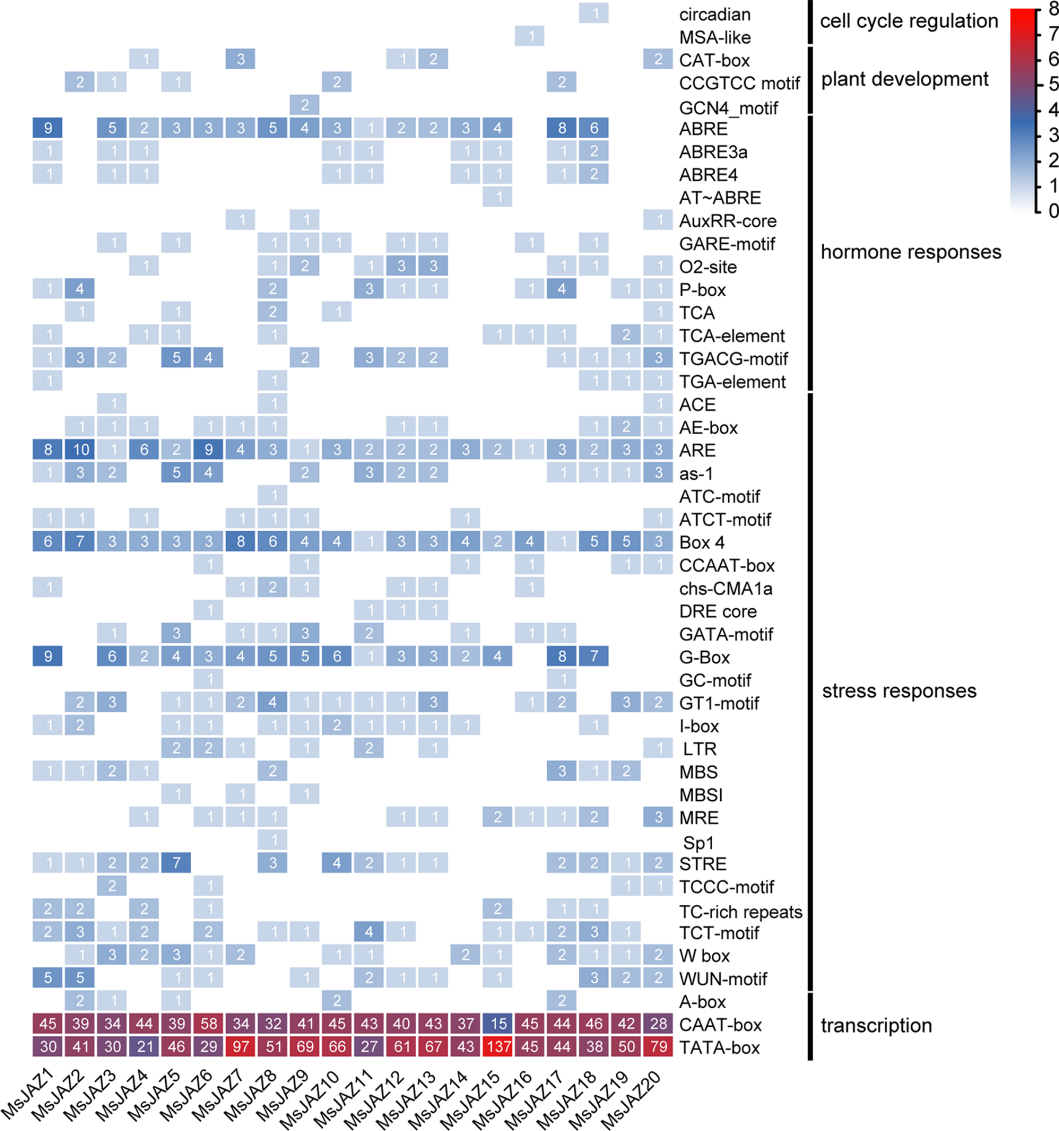
Prediction of *cis*-acting elements in *MsJAZ* gene promoters. These *cis*-acting elements were identified by PlantCARE. The values in the figure represent logarithmic conversions, and low-to-high ranges of element numbers are indicated by the colours white and red

Identification and analysis of the *cis*-acting elements in the upstream sequences of genes allow us to determine whether these genes have the potential to participate in growth, development, and response to biotic or abiotic stresses. In this study, the *cis*-acting elements in the 2000 bp upstream regions of *MsJAZ* genes were identified and analysed via PlantCARE (Fig. 7). Many *cis*-acting elements were identified in these sequences, and based on their functions, they were classified into five categories: cell cycle regulation, plant development, hormone responses, stress responses, and transcription regulation. There were 2, 3, 12, 26, and 3 *cis*-acting elements associated with the cell cycle, development, hormones, stress, and transcription, respectively. A number of *cis*-regulatory elements, including AE-boxes, GATA motifs, GT1 motifs, LTRs, MBSs, and TC-rich repeats, have been demonstrated to be involved in the response to various abiotic and biotic stresses. In our study, we found that 11, 9, 15, 7, 8, and 7 *MsJAZ* genes contained AE-boxes, GATA motifs, GT1 motifs, LTRs, MBSs, and TC-rich repeats, respectively. In addition, all *MsJAZ* genes contained multiple CAAT-box and TATA-box *cis*-acting elements, indicating their potential significance in transcriptional regulation.

### Expression pattern analysis of *MsJAZ* genes in different tissues

**Fig. 8:**
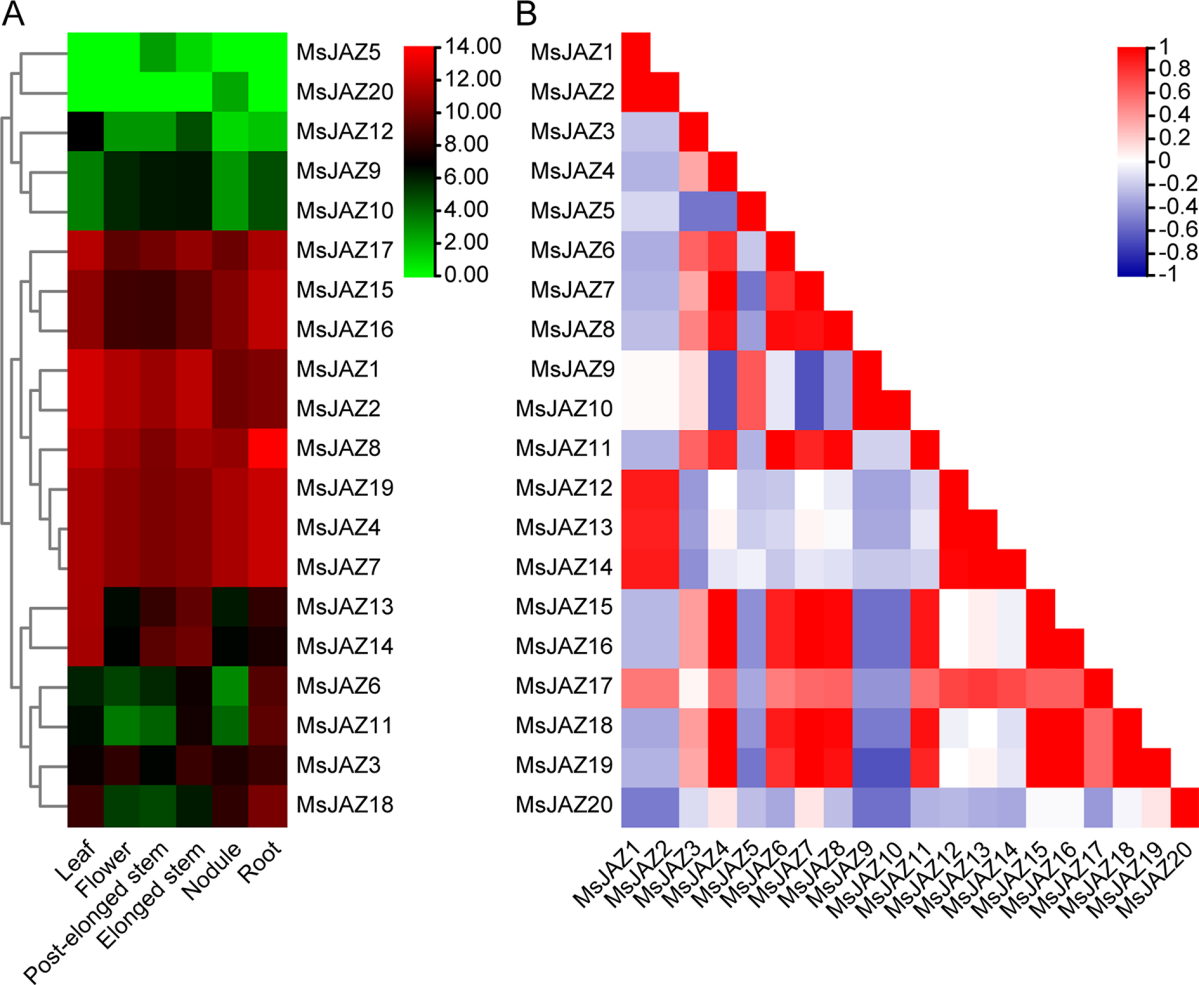
Expression analysis of alfalfa *JAZ* genes in six tissues. (**A**) Heatmap showing the differential expression of *MsJAZ* genes in six tissues. The values represent logarithmic conversions. Green and red indicate low and high *MsJAZ* gene expression, respectively. (**B**) Correlated heatmaps of the expression patterns in six tissues. The correlations are depicted using the colours blue and red, representing negative and positive correlations, respectively

The expression of *JAZ* genes in various tissues of alfalfa reflects their specific functions in those tissues, providing the basis for the important role that *JAZ* genes play in the growth of alfalfa. Consequently, the expression levels of the 20 *MsJAZ* genes were determined across six different tissues, namely, leaves, flowers, pre-elongated stems, elongated stems, roots, and nodules. TBtools software was used to visualize the expression patterns of the *MsJAZ* genes, and the results showed that the expression profiles of the *MsJAZ* genes could be classified into three types (Fig. 8A). The expression of *MsJAZ5*, *MsJAZ9*, *MsJAZ10*, *MsJAZ12*, and *MsJAZ20* was lowest in all tissues. *MsJAZ1*, *MsJAZ2*, *MsJAZ4*, *MsJAZ7*, *MsJAZ8*, *MsJAZ15*, *MsJAZ16*, *MsJAZ17*, and *MsJAZ19* exhibited the highest expression levels in all tissues, indicating their significant role across various tissues. *MsJAZ3*, *MsJAZ6*, *MsJAZ11*, *MsJAZ13*, *MsJAZ14*, and *MsJAZ18* exhibited high expression in some tissues and low expression in other tissues. Additionally, correlation analysis revealed that the expression patterns of most *MsJAZ* genes were positively correlated, but only a small subset of *MsJAZ* genes exhibited a significant direct correlation (Fig. 8B).

### Expression pattern analysis of *MsJAZ* genes in response to salt stress 

**Fig. 9:**
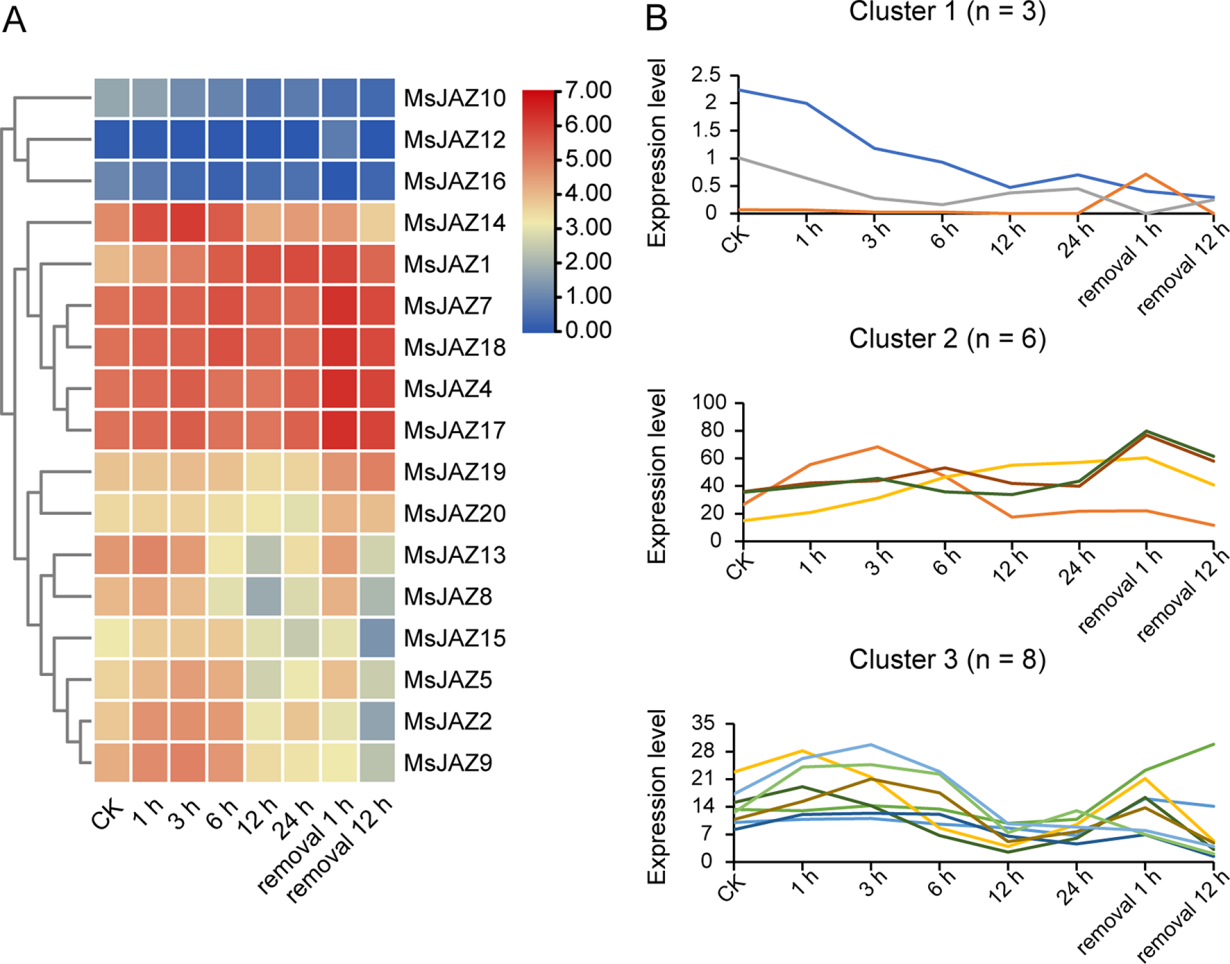
Expression pattern analysis of alfalfa *JAZ* genes under continuous salt treatment. (**A**) The heatmap shows that the expression levels of the *MsJAZ* gene in the root tips changed at 0 (CK), 1, 3, 6, 12, and 24 h, as well as at 1 and 12 h after continuous salt treatment. The values in the figure are logarithmic conversions. Blue and red indicate low and high *MsJAZ* gene expression, respectively. (**B**) Expression trend analysis of *MsJAZ* genes at 0 (CK), 1, 3, 6, 12, and 24 h of salt treatment and at 1 and 12 h after continuous salt treatment

To determine the dynamic changes in the gene expression of *MsJAZ* genes under salt stress treatment, we retrieved the expression levels of *MsJAZ* genes under salt stress through BLASTN. These transcriptome data were obtained after continuous salt treatment of alfalfa root tips for 0, 1, 3, 6, 12, and 24 h, as well as at 1 and 12 h after stress removal. Seventeen *MsJAZ* genes were found in this transcriptome dataset, and the remaining three genes were not found. TBtools software was used to construct a heatmap of the expression patterns of these 17 *MsJAZ* genes (Fig. 9A), and the R software package Mfuzz was used to analyse the time-related dynamic expression patterns of the *MsJAZ* genes (Fig. 9B). The expression trends of most of the *MsJAZ* genes were similar; with increasing stress duration, the expression levels increased and then decreased, and after the stress was removed, the levels immediately increased and then decreased, but the fold increase and response time differed. In addition, three clusters were formed by analysing the expression patterns of these 17 *MsJAZ* genes. The number of *MsJAZ* genes in Cluster 3 was the greatest (*n* = 8), followed by Cluster 2 (*n* = 6), and Cluster 1 contained the fewest (*n* = 3). The response patterns of the genes in Cluster 2 (*MsJAZ1*, *MsJAZ4*, *MsJAZ7*, *MsJAZ14*, *MsJAZ17*, and *MsJAZ18*) to salt stress were similar, and their expression levels were close and significantly greater than those of other *MsJAZ* genes. The genes in Cluster 1 (*MsJAZ10*, *MsJAZ12*, and *MsJAZ16*) had similar expression profiles, and their expression levels were close and significantly lower than those of the other clusters. The expression levels of the genes in Cluster 3 (*MsJAZ2*, *MsJAZ5*, *MsJAZ8*, *MsJAZ9*, *MsJAZ13*, *MsJAZ15*, *MsJAZ19*, and *MsJAZ20*) were moderate, and the changes were not significant.

### RT‒qPCR validation

**Fig. 10:**
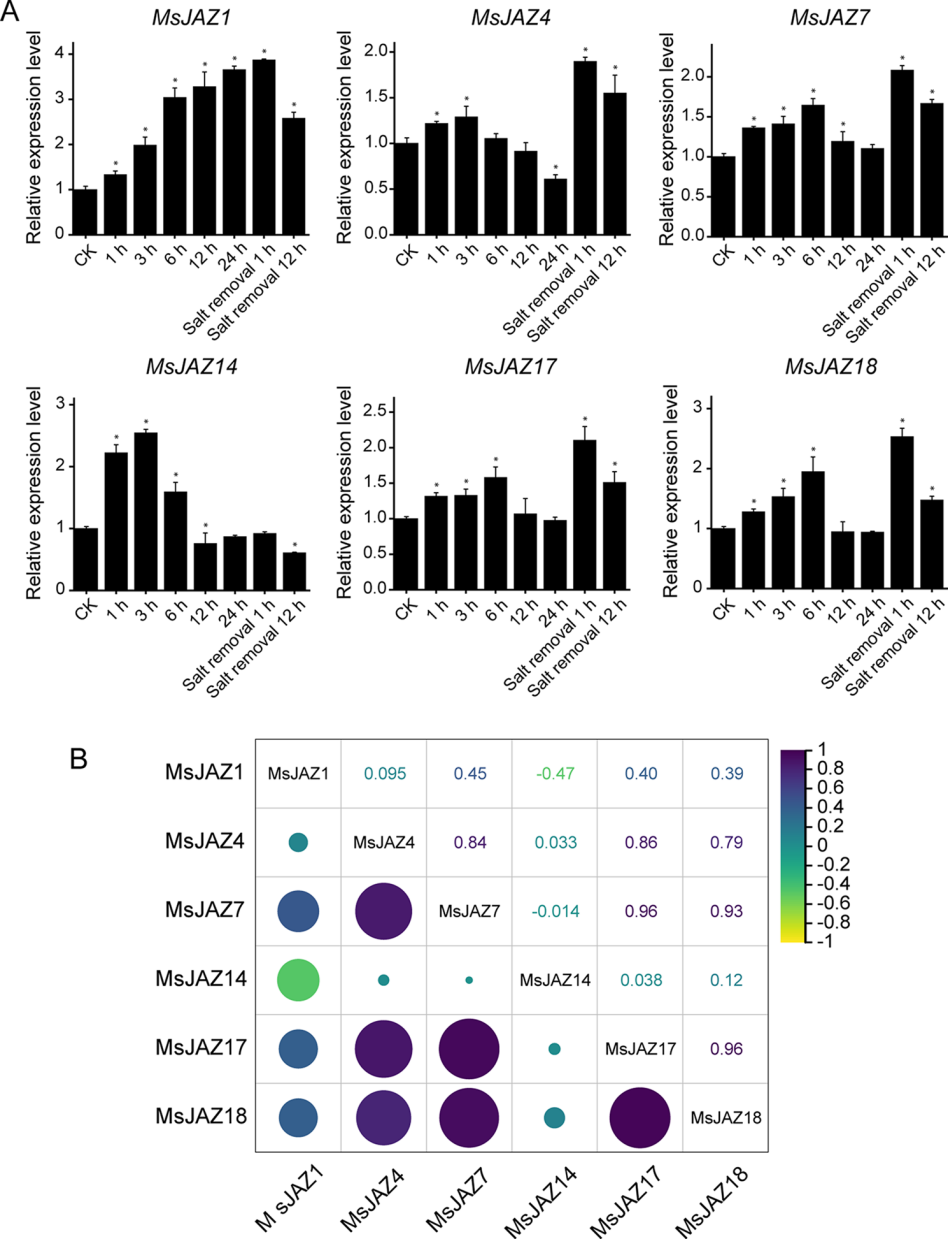
Relative expression levels of six selected *MsJAZ* genes under salt treatment. (**A**) The relative expression levels of six *MsJAZ* genes under salt treatment for different durations determined by RT‒qPCR. The data in the figure are the means ± SDs of three replicates. Asterisks indicate significant differences, and *p* < 0.05 (*) indicates extreme significance. (**B**) The correlation of the gene expression patterns of the six *MsJAZ* genes. Black and yellow indicate positive and negative correlations, respectively

To further study the response of the *MsJAZ* gene to salt stress, we selected six *MsJAZ* genes (*MsJAZ1*, *MsJAZ4*, *MsJAZ7*, *MsJAZ14*, *MsJAZ17*, and *MsJAZ18*) whose expression was positively induced by salt stress for RT‒qPCR experiments, and the results are shown in Fig. 10A. The expression patterns of all the selected *MsJAZ* genes were found to be consistent with the RNA-Seq data, indicating that the expression of these genes increased under salt stress. Among these genes, the expression of *MsJAZ1* continued to increase and peaked at 1 h after removal of salt treatment, which was in line with the RNA-Seq data. The expression trends of the other five genes were similar; they all increased with increasing salt stress, decreased after reaching the peak value, and then peaked a second time at 1 h after salt removal, but they first peaked at different times. Overall, the expression trend of all *MsJAZ* genes in the RT‒qPCR assay was in line with the RNA‒Seq results, but the magnitudes of the changes in the RNA‒Seq and RT‒qPCR results differed. In addition, except for *MsJAZ1*/*MsJAZ14* and *MsJAZ7*/*MsJAZ14*, which were negatively correlated, the other genes were directly positively correlated (Fig. 10B).

### Subcellular localization of the MsJAZ1 protein

**Fig. 11:**
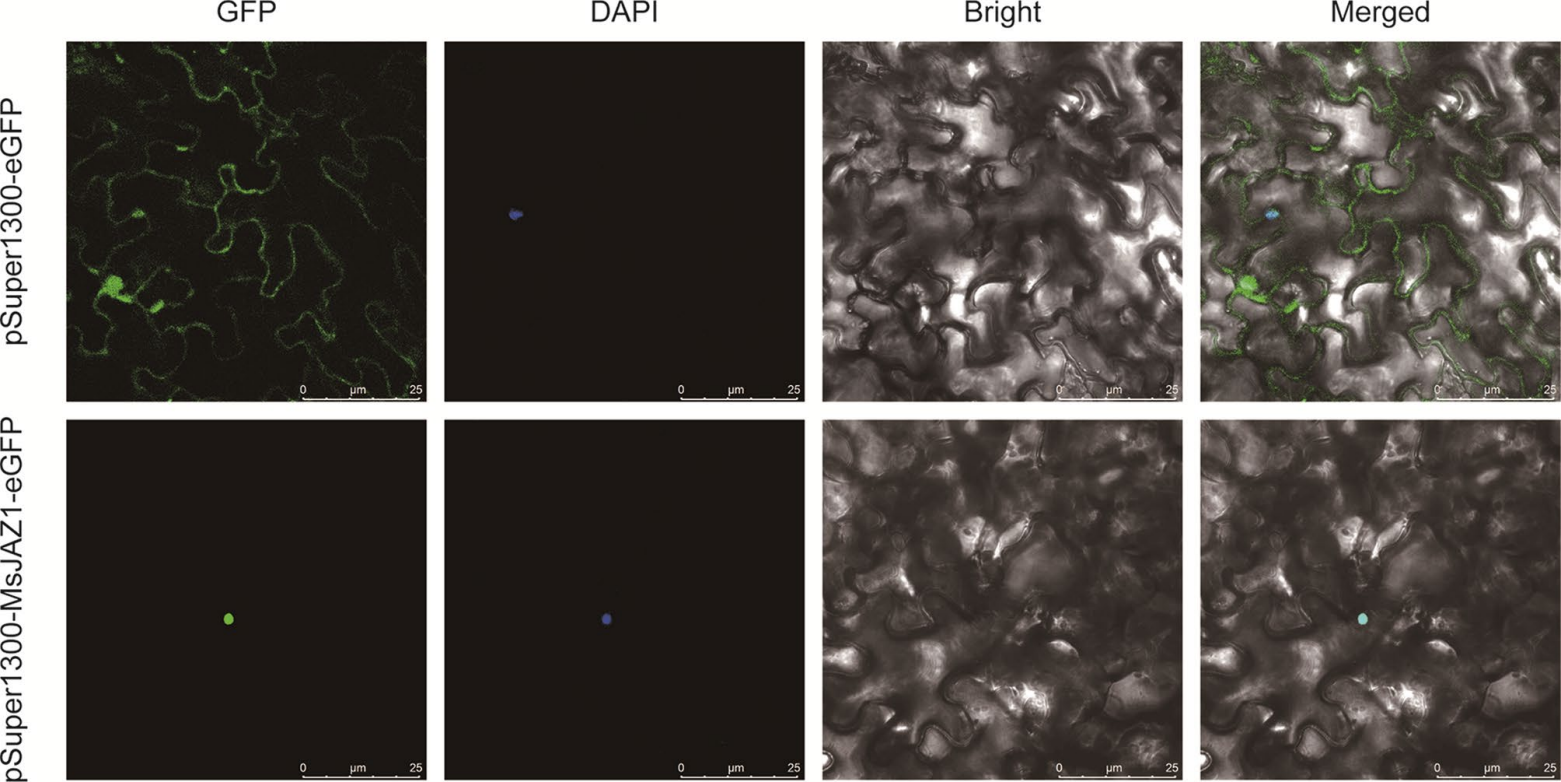
Subcellular localization of the MsJAZ1 proteins. Images from left to right represent green fluorescent protein (GFP), DAPI, bright field and an overlay (GFP, DAPI, and bright field) from the same sample

The subcellular localization prediction of the selected MsJAZ1 protein by WoLF PSORT revealed its localization in the nucleus (Table S3). To validate this prediction and further investigate the genes significantly induced by salt stress, we performed transient expression experiments in tobacco leaves. The results showed that the GFP fluorescence overlapped with DAPI staining, indicating its localization in the nucleus, consistent with the predicted results (Fig. 11).

## Discussion

Jasmonoyl-L-isoleucine (JA-Ile) is a crucial signalling molecule that plays a significant role in regulating various aspects of plant growth, development, and defence responses [23, 50, 51]. JAZ proteins are key components of the JA pathway and can negatively regulate signal transduction of JAs. JAZ proteins inhibit the activity of DNA-binding transcription factors that regulate the transcription of JA response genes [36]. To improve the adaptability of plants to adverse conditions, plants need to inhibit growth and other vital activities, and the expression of the *JAZ* gene can reduce the production of JA, which can regulate metabolic processes [35]. In addition, with the continuous development of biotechnology, the genome-wide identification of *JAZ* genes has been performed for several plants, such as *Arabidopsis* [25, 30], rice [52], maize [53], *Sorghum bicolor* [38], common Fig. [54], bread wheat [55], tomato [56], tea [57], and soybean [58]. However, no comprehensive identification or analysis of the alfalfa *JAZ* gene family has been reported.

Here, 20 *JAZ* genes were identified in the XinJiangDaYe genome. The number of *JAZ* genes in alfalfa was greater than that in *Arabidopsis* (*n* = 13) [25, 30], rice (*n* = 15) [52], maize (*n* = 16) [53], *Sorghum bicolor* (*n* = 17) [38], common fig (*n* = 10) [54], bread wheat (*n* = 14) [55], tomato (*n* = 9) [56], and tea (*n* = 13) [57] but less than that in soybean (*n* = 33) [58]. The MsJAZ proteins differed in length, molecular weight (MW), and theoretical isoelectric point (PI) but exhibited similar instability indices (IIs) and grand average hydropathicity indices (GRAVYs). Through subcellular localization prediction, we found that all the MsJAZ proteins were located in the nucleus, while the MsJAZ5, MsJAZ9, and MsJAZ10 proteins may also be located in the cell membrane. The subcellular localization experiment of MsJAZ1 protein was conducted, and the results were consistent with the prediction. The protein properties and subcellular localization predictions of MsJAZ proteins were similar to those in other plants [31, 39]. According to the GO analysis results, all *MsJAZ* genes were associated with biological processes and molecular functions, indicating that they are important for the growth and development of alfalfa.

Phylogenetic analysis revealed that the 20 *MsJAZ* genes could be divided into five subfamilies based on their evolutionary relationships with *Arabidopsis JAZ* genes. The results obtained via the NJ, ML, ME, and UPGMA methods were similar, but those obtained via the NJ method were more similar to those of the *Arabidopsis JAZ* gene classification [25, 30]. The clustering of *JAZ* genes from alfalfa and *M. truncatula* in the same branch was the most prominent, indicating that the genetic relationship of alfalfa with *M. truncatula* was closer than that with *Arabidopsis* or rice. Genes located on the same evolutionary branch may have the same function, so the same function of these genes can be predicted based on homology and phylogenetic analysis with other species [59]. AtJAZ1 [60] and OsJAZ9 [33] have been demonstrated to be associated with the regulation of salt stress, and thus, the MsJAZ proteins with AtJAZ1 and OsJAZ9 in group I may regulate salt stress. All *MsJAZ* genes contained three to eight exons and two to eight motifs, and the *MsJAZ* genes in the same subfamily had similar gene structures and motifs, indicating that these genes in the same subgroup may have subgroup-specific functions. All *MsJAZ* genes had motif 1 and motif 2. Motif 1 and motif 2 were identified as the TIFY domain and JAS domain, respectively, according to the NCBI-CDD results, and their positions were consistent with those in previous studies [26, 30]. Many of the 3D structures of MsJAZ proteins are similar, and therefore, MsJAZ proteins may have similar functions [61].

Gene replication plays a very important role in the evolutionary expansion of all gene families in plants [62]. In our study, 8 pairs of segmental duplications but no tandem duplications were detected in alfalfa, indicating that segmental duplications rather than tandem duplications played an important role in the evolutionary expansion of *MsJAZ*. Most Ka/Ks ratios of duplicated gene pairs were less than one, suggesting that the *JAZ* genes mainly evolved under the effect of purifying selection in alfalfa [63]. Identification of homologous genes between different species by collinearity analysis is helpful for understanding the role of genes in plant evolution [64]. Therefore, we analysed and compared the homologous genes of alfalfa with those of *A. thaliana*, *M. truncatula*, and *O. sativa*. The results showed that there were more homologous gene pairs between alfalfa and *M. truncatula* than between *A. thaliana* and *O. sativa*, suggesting that the genetic background of autotetraploid alfalfa is more similar to that of *M. truncatula*. A similar phenomenon has been found in cassava [65]. In addition, we found that *MsJAZ8* and *MsJAZ17* were collinear in all the plants, indicating that these genes played a vital role in the evolution of *JAZ* genes.

*Cis*-acting element analysis can help predict the possible roles of genes in biotic and abiotic stress signal responses [66]. Many stress-related *cis*-acting elements, such as ABREs, anaerobic induction essential elements (AREs), low-temperature response elements (LTRs), and MYB-binding sites involved in drought responses (MBSs), have been identified in the promoter sequences of MsJAZ genes, and these elements were also found in the *JAZ* gene family members of sweet potato [37]. These *cis-*acting elements have been reported to be associated with stress. For example, ABREs can interact with upstream transcription factors to activate ABA-responsive genes, thereby improving plant stress resistance [67]. In addition, these stress-related *cis*-acting elements were also present in the promoter regions of stress response genes, such as *MsDof* [68], *MsWRKY* [69], and *MsMYB* [70]. The *MsJAZ* genes had these *cis*-acting elements, suggesting that they have the potential to respond to salt stress.

JA can promote plant senescence and inhibit seedling growth, while the JAZ protein, an important member of the JA signal transduction pathway, also plays an important role in plant growth and development [10, 11]. In *Arabidopsis*, JAZ4 functions as a negative regulator of ethylene (ET) signalling and auxin signalling in the root tissues above the apex, but in the root apex, JAZ4 might act as a positive regulator of auxin signalling, potentially independent of ethylene signalling, and these distinct roles have an impact on root growth and development [71]. In soybeans, GmJAZ3 interacts with GmRR18a and GmMYC2a to regulate seed size and weight [72]. In different tissues, the regulatory function of genes mainly depends on their expression levels [73]. In our study, the *MsJAZ* genes were differentially expressed in different tissues, suggesting differences in their functions in plant growth and development. *MsJAZ1*, *MsJAZ2*, *MsJAZ4*, *MsJAZ7*, *MsJAZ8*, *MsJAZ15*, *MsJAZ16*, *MsJAZ17*, and *MsJAZ19* were significantly more highly expressed than other *MsJAZ* genes in all tissues, indicating that these genes may be important at all stages of plant development. Moreover, *MsJAZ13* and *MsJAZ14* were highly expressed in leaves, which indicated that these *MsJAZ* genes could control leaf development.

JA is essential for the growth-defence balance of plants, as JA can both promote defence and inhibit plant growth [74]. The related role of *JAZ* genes in salt tolerance has been reported in many plants. For instance, GhJAZ1 can promote the expression of resistance-related genes and root growth, increasing the vitality, height, fresh weight, and fruiting rate of transgenic plants, thereby enhancing the activity of upland cotton (*Gossypium hirsutum*) plants under salt stress [75]. Overexpression of apple *MdJAZ2* enhanced the salt tolerance of *Arabidopsis* [76]. PnJAZ1, through crosstalking with the abscisic acid signalling pathway, enhances the growth of *Pohlia nutans* plants under salt stress [77]. In this study, a total of 17 *MsJAZ* genes were identified based on our RNA-Seq data. Overall, the expression levels of the majority of these *MsJAZ* genes were upregulated under salt treatment. These findings suggested that most *MsJAZ* genes likely play a positive regulatory role in the response to salt stress. Additionally, we performed RT‒qPCR experiments on six genes that were found to be significantly induced by salt stress according to the RNA-Seq analysis. We observed a significant increase in their relative expression levels under salt stress, which was consistent with the expression trends observed in the RNA-Seq analysis. However, the fold change in the expression levels of these selected genes differed between the RNA-Seq analysis and the RT‒qPCR experiment. This discrepancy may be attributed to the biological diversity among different individuals of alfalfa [78].

## Conclusions

In this study, a total of 20 *JAZ* gene family members were identified in the autotetraploid cultivated alfalfa genome. Several aspects of these genes were investigated, including their physicochemical properties, evolutionary relationships, gene structures, protein motif compositions, 3D protein structures, gene duplication events, chromosomal distribution, and *cis*-acting elements, as well as their expression levels in different tissues and under salt stress conditions. In addition, expression analysis revealed that *MsJAZ1*, *MsJAZ4*, *MsJAZ7*, *MsJAZ14*, *MsJAZ7* and *MsJAZ18* significantly responded to salt stress. In conclusion, our study is the first to provide a comprehensive identification and analysis of alfalfa *JAZ* gene family members at the autotetraploid level. These findings establish a strong foundation for future research on the function and molecular mechanisms of *JAZ* genes in the salt stress response of autotetraploid cultivated alfalfa.

### Electronic supplementary material

Below is the link to the electronic supplementary material.

Supplementary Material 1

Supplementary Material 2

Supplementary Material 3

Supplementary Material 4

Supplementary Material 5

Supplementary Material 6

Supplementary Material 7

Supplementary Material 8

Supplementary Material 9

## Data Availability

The draft genome data of autotetraploid cultivated alfalfa was obtained from (figshare.com/projects/whole_genome_sequencing_and_assembly_of_Medicago_sativa/66380). Genome-wide transcriptome data of different alfalfa tissues were acquired from the MODMS (modms.lzu.edu.cn). All transcriptome sequencing data used in this study were available in NCBI SRA (www.ncbi.nlm.nih.gov/sra/): SRR7160313-SRR7160357 (salt treatment).

## Abbreviations

JAZ: Jasmonate-ZIM
HMM: Hidden Markov Model
GO: Gene Ontology
JA: Jasmonic Acid
JA-Ile: Jasmonoyl-Isoleucine
Jas: Jasmonates
MeJA: Methyl Jasmonate
Chr: Chromosome
