# Supplementary figures and images for: Genome-wide identification of JAZ gene family members in autotetraploid cultivated alfalfa (Medicago sativa subsp. sativa) and expression analysis under salt stress
Source: BMC Genomics. 2024 Jun 26;25:636. doi: 10.1186/s12864-024-10460-6 (PMC11201308; doi:10.1186/s12864-024-10460-6)

Fig. S1
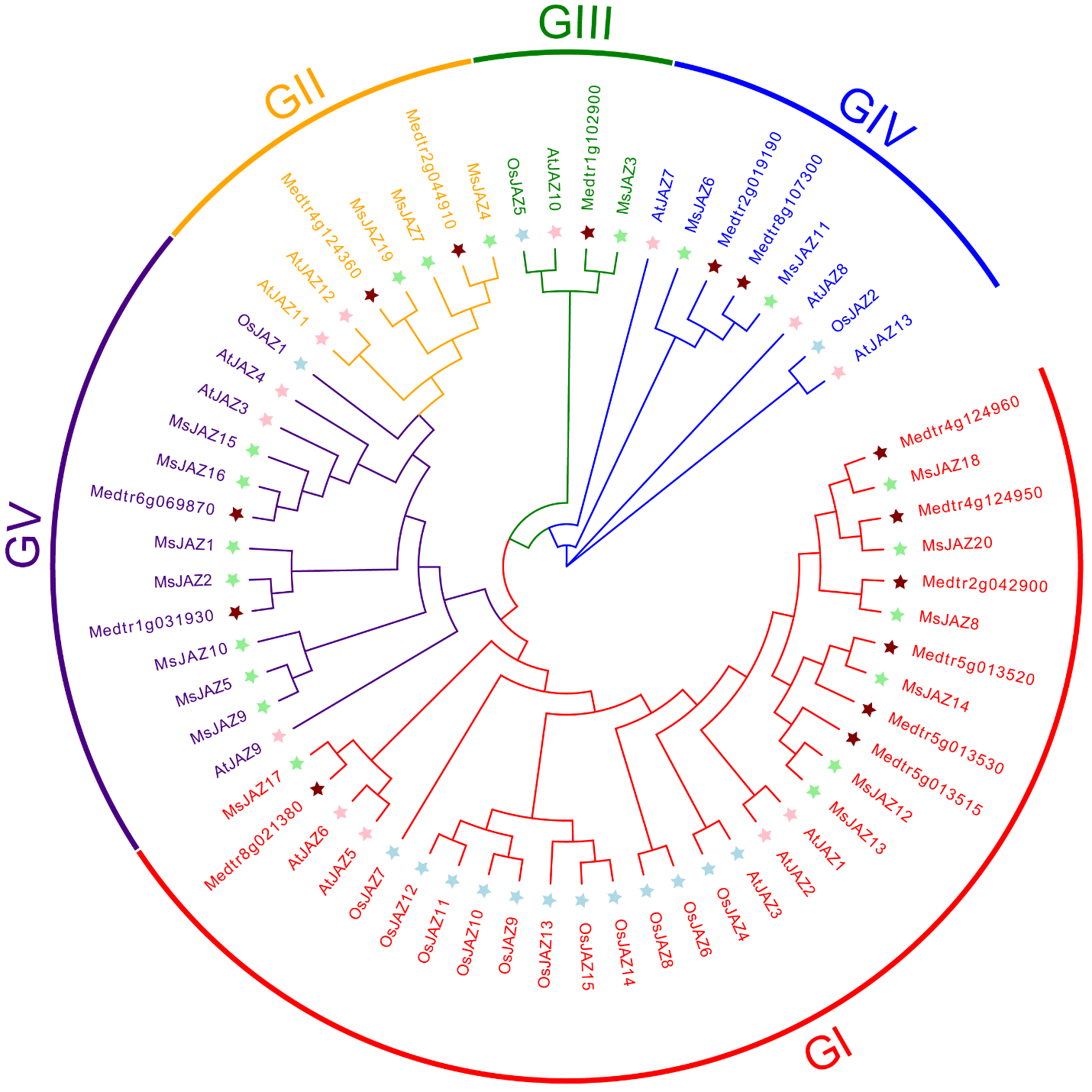


Fig. S2


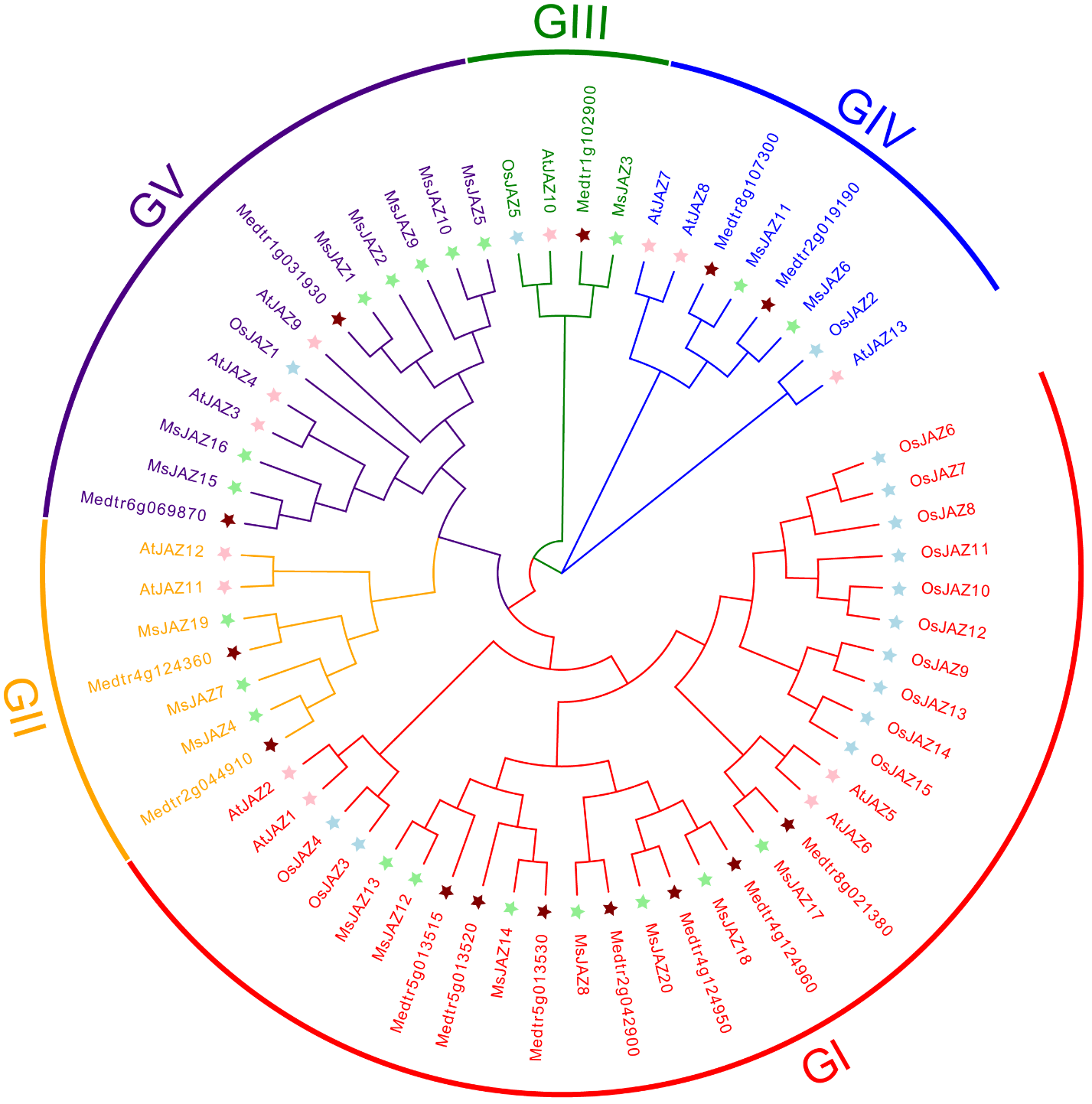


Fig. S3


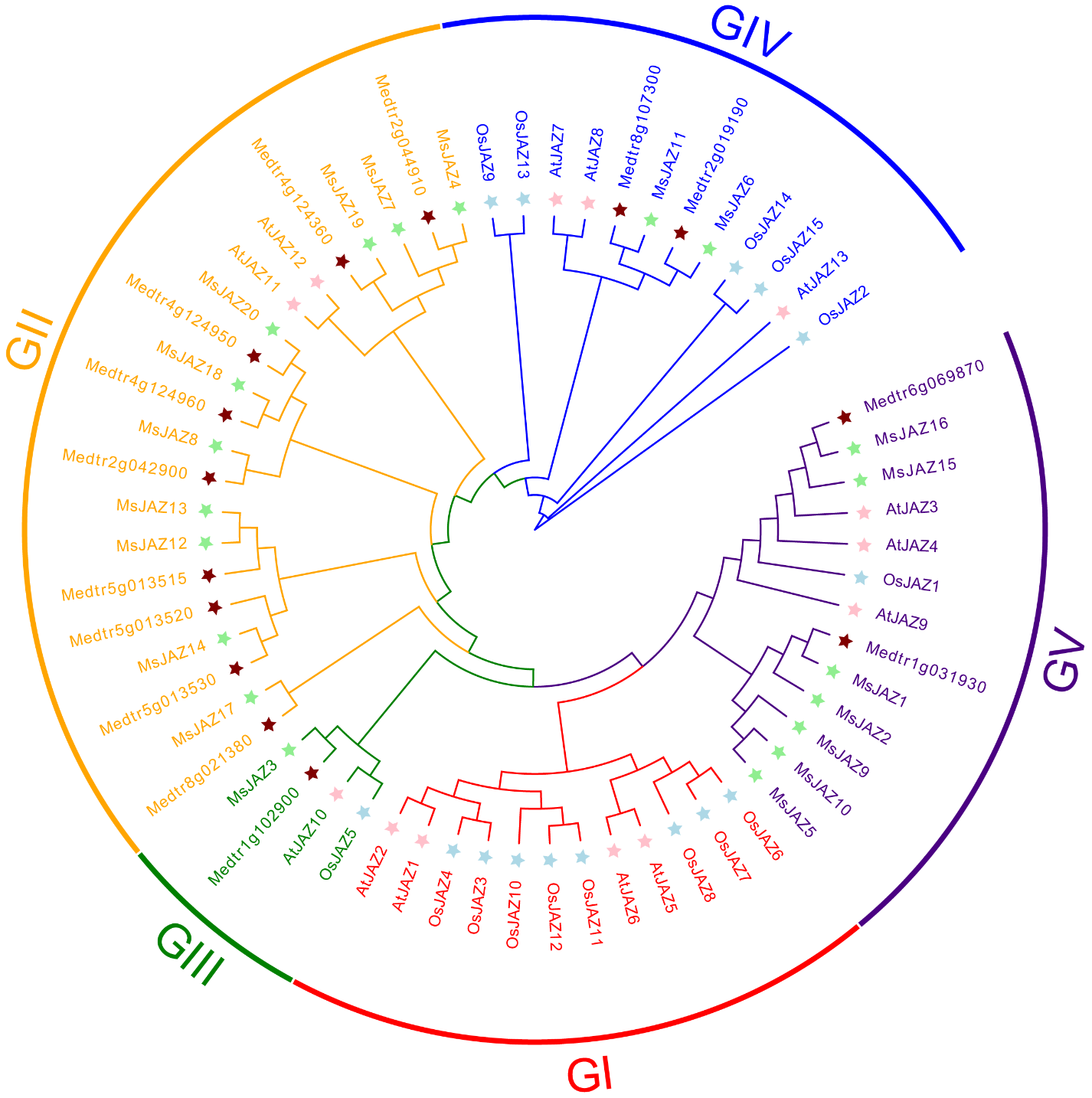

Supplement: Supplementary file 1 — Supplementary Material 1 [file 12864_2024_10460_MOESM1_ESM.docx]
